# Supplementary material for: Brain Metabolic Profile after Intranasal vs. Intraperitoneal Clomipramine Treatment in Rats with Ultrasound Model of Depression
Source: Int J Mol Sci. 2021 Sep 4;22(17):9598. doi: 10.3390/ijms22179598 (PMC8431753; doi:10.3390/ijms22179598)
Supplement: Supplementary file 1 [file ijms-22-09598-s001.zip › ijms-1359011-supplementary.pdf]

International Journal of
Molecular Sciences

Supplementary files

Principal component analysis

Hippocampus

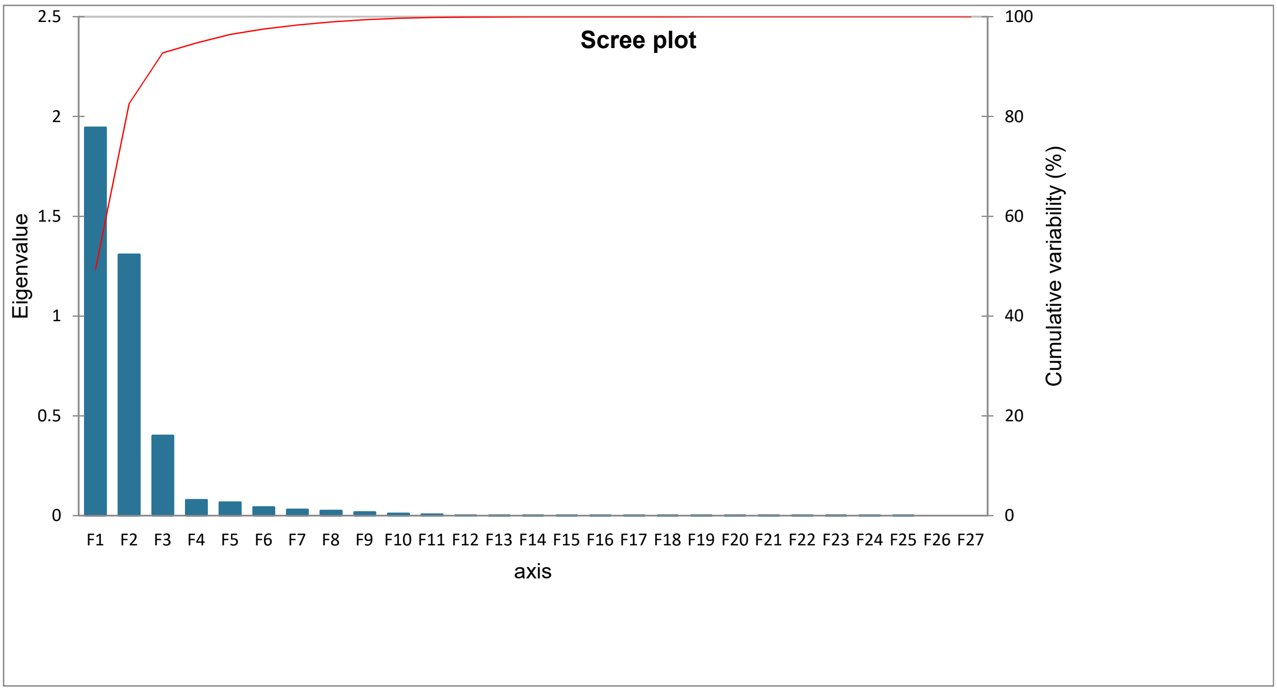

Figure S1. Scree-plot of PCA of number factors

Frontal cortex

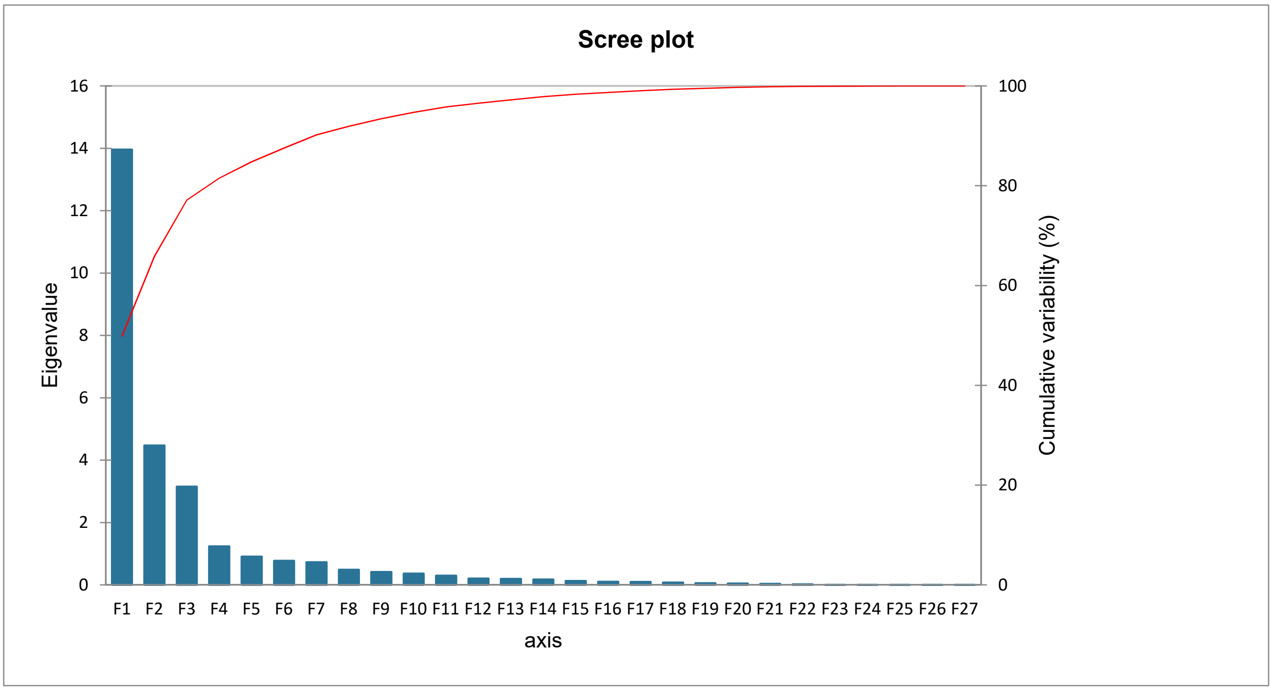

Figure S2. Scree-plot of PCA number factors in frontal cortex

Heatmaps

Frontal cortex

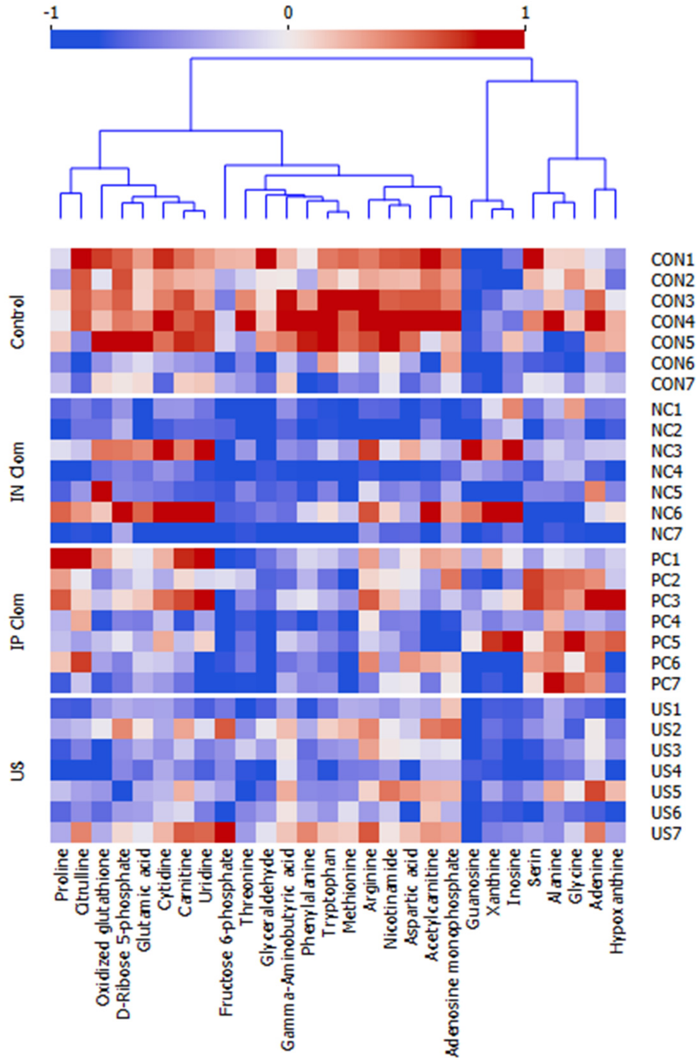

Figure S3. Clustered heatmap of metabolites in the frontal cortex of individual rat

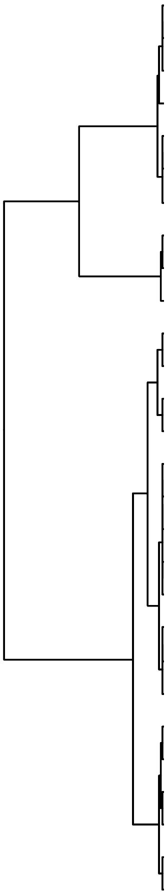

Metabolites in the frontal cortex	Control	IN Clom	IP Clom	US	Mann-Whitney pairwise comparison *p* -value					
					Control - US	Control - IN Clom	Control - IP Clom	IN Clom - US	IP Clom - US	IN Clom - IP Clom
Citrulline	0.44	0.33	0.45	0.33						
Serin	0.31	0.23	0.32	0.24		0.020				
Hypoxanthine	0.13	0.11	0.13	0.10		0.003	0.024	0.026	0.038	
Adenine	0.09	0.07	0.09	0.08	0.038	0.002	0.011			
Alanine	0.11	0.08	0.14	0.09					0.002	0.002
Proline	0.85	0.70	0.95	0.69						
Glycine	0.00	0.00	0.00	0.00	0.026	0.004	0.007			
Guanosine	0.00	0.00	0.00	0.00					0.004	0.012
Xanthine	0.02	0.04	0.04	0.02						
Inosine	0.01	0.03	0.02	0.01					0.026	0.026
D-Ribose 5-phosphate	0.03	0.02	0.01	0.01						
Oxidized glutathione	0.11	0.08	0.07	0.06						
Uridine	0.03	0.02	0.02	0.02				0.026		
Cytidine	1.17	0.92	1.01	0.89						
Aspartic acid	0.39	0.26	0.33	0.31	0.007	0.004			0.003	0.002
Phenylalanine	0.31	0.17	0.23	0.23		0.002		0.020		
Arginine	0.20	0.15	0.18	0.17		0.038				0.038
Carnitine	1.12	0.83	0.92	0.94		0.017	0.017			
Nicotinamide	4.13	2.81	3.26	3.42	0.017	0.004	0.026			
Tryptophan	0.58	0.31	0.33	0.37		0.002	0.002	0.026	0.011	
Threonine	1.39	0.90	0.98	0.97	0.002		0.017			
Glutamic acid	4.76	3.40	3.83	3.63	0.026					
Adenosine monophosphate	2.84	1.61	2.06	2.40		0.017				0.038
Gamma-Aminobutyric acid	1.71	0.74	0.98	1.41	0.011		0.026			
Methionine	0.09	0.04	0.04	0.06		0.001	0.002	0.001	0.004	
Glyceraldehyde	0.02	0.00	0.00	0.01	0.007		0.011			
Fructose 6-phosphate	0.00	0.00	0.00	0.00						
Acetylcarnitine	0.53	0.40	0.39	0.50	0.017				0.017	

Figure S4. Clustered mean frontal cortex concentration of metabolites in animal groups with pairwise comparison between groups

Hippocampus

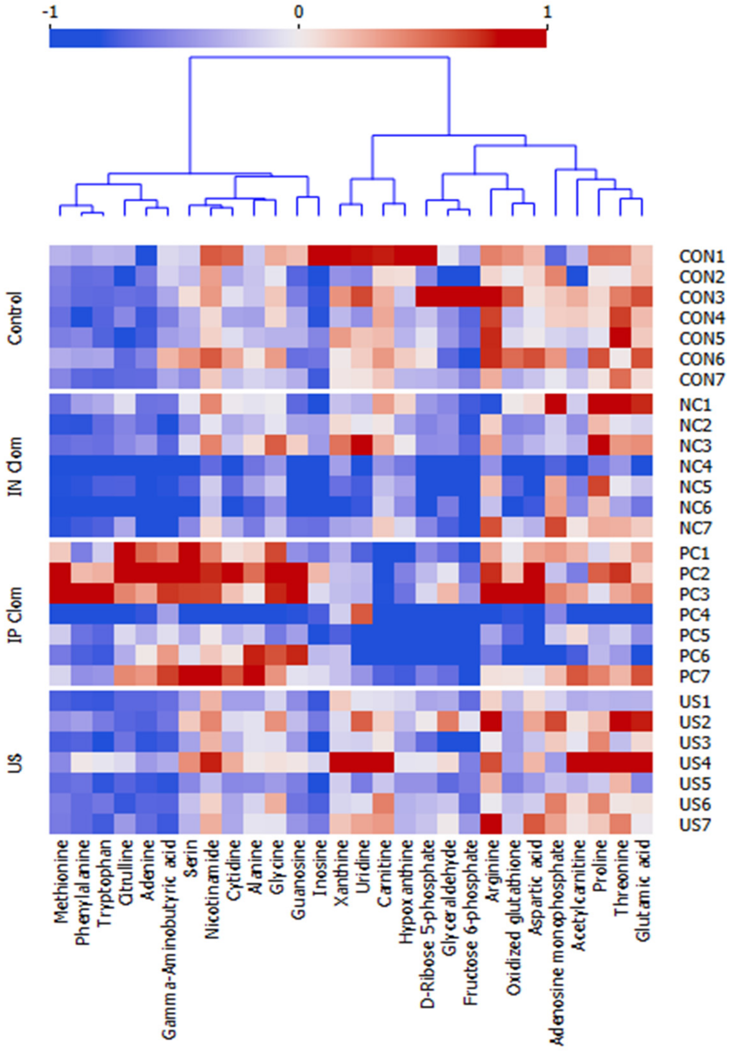

Figure S5. Clustered heatmap of metabolites in the frontal cortex of individual rat

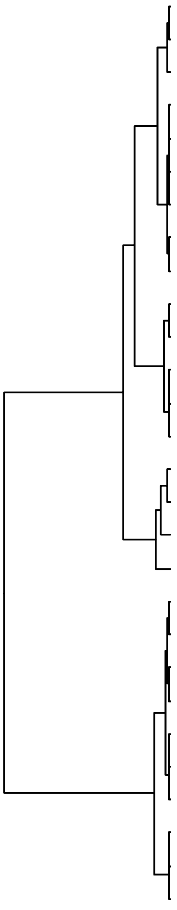

Metabolites in the hippocampus	Control	IN Clom	IP Clom	US	Mann-Whitney pairwise comparison *p*-value					
					Control - US	Control - IN Clom	Control - IP Clom	IN Clom - US	IP Clom - US	IN Clom - IP Clom
Aspartic acid	0.38	0.35	0.35	0.21		0.007		0.011		
Nicotinamide	4.13	3.97	3.88	3.11						
Oxidized glutathione	0.20	0.14	0.17	0.08	0.011	0.017				
Glutamic acid	5.00	4.79	4.12	3.41						
Uridine	0.05	0.05	0.04	0.03						
Threonine	1.14	1.19	0.95	0.82						
Xanthine	0.13	0.14	0.10	0.07					0.001	
Arginine	0.21	0.20	0.16	0.14		0.026				
D-Ribose 5-phosphate	0.06	0.05	0.03	0.02		0.002	0.011	0.011		
Hypoxanthine	0.16	0.13	0.07	0.11			0.001		0.001	0.001
Carnitine	2.15	2.20	0.64	1.56			0.001		0.001	0.001
Adenosine monophosphate	1.95	1.95	1.41	1.42						
Fructose 6-phosphate	0.01	0.01	0.00	0.00				0.011		
Proline	1.05	1.05	0.79	0.85						
Acetylcarnitine	0.89	1.14	0.89	0.70				0.029		
Glyceraldehyde	0.02	0.03	0.02	0.02						
Guanosine	0.00	0.01	0.01	0.00						
Glycine	0.00	0.00	0.00	0.00						
Cytidine	1.85	1.60	1.88	1.10						0.049
Alanine	0.15	0.14	0.16	0.11						
Serin	0.37	0.35	0.44	0.23						
Phenylalanine	0.34	0.34	0.45	0.20						
Methionine	0.11	0.09	0.18	0.05		0.001		0.017		0.017
Tryptophan	0.54	0.49	0.78	0.33						
Gamma-Aminobutyric acid	2.72	1.50	3.69	0.95	0.007	0.001	0.017		0.001	0.001
Inosine	0.04	0.05	0.07	0.04						0.038
Citrulline	0.59	0.66	1.11	0.54			0.017			0.038
Adenine	0.11	0.10	0.19	0.09			0.017		0.017	0.004

Figure S6. Clustered mean hippocampus concentration of metabolites in animal groups with pairwise comparison between groups

Table S1**.** Results from the pathway analysis; Total - the total number of compounds in the pathway; Hits - the actually matched number from the user uploaded data; The p - the original p-value calculated from the enrichment analysis; Holm p - the p-value adjusted by Holm-Bonferroni method; FDR - the p-value adjusted using False Discovery Rate; Impact - the pathway impact value calculated from pathway topology analysis.

Pathway Name	Total	Hits	p	-log(p)	Holm p	FDR	Impact
**A. Metabolic pathways in frontal cortex related to depression-like behaviors induced by US stress.**							
Tryptophan metabolism	41	1	4.4473E-5	4.3519	0.0012	0.0012	0.1431

Aminoacyl-tRNA biosynthesis	48	10	9.3534E-4	3.029	0.0243	0.0026	0.1667
Alanine, aspartate and glutamate metabolism	28	4	0.0011	2.9426	0.0285	0.0026	0.5072
Arginine and proline metabolism	38	4	0.0011	2.9414	0.0285	0.0026	0.2455
Arginine biosynthesis	14	4	0.0012	2.9381	0.0285	0.0026	0.4213
D-Glutamine and D-glutamate metabolism	6	1	0.0012	2.9331	0.0285	0.0026	0.5
Pentose phosphate pathway	21	1	0.0052	2.2855	0.0777	0.0108	0.185
Nicotinate and nicotinamide metabolism	15	2	0.0061	2.2133	0.0857	0.0118	0.1943
Glycine, serine and threonine metabolism	34	2	0.0344	1.4635	0.432	0.058	0.2307
**B. Metabolic pathways in hippocampus related to antidepressant effect of intraperitoneal clomipramine**							
Purine metabolism	66	7	0.0216	1.665	0.6056	0.4853	0.1305
